# The prevalence of RT 245 codon polymorphisms and its association with duration of infection among HIV-1 patients in Serbia

**DOI:** 10.1186/1471-2334-14-S4-P4

**Published:** 2014-05-29

**Authors:** Valentina Nikolic, Dubravka Salemovic, Djordje Jevtovic, Ivana Pesic-Pavlovic, Sonja Zerjav, Marina Siljic, Jovan Ranin, Maja Stanojevic

**Affiliations:** 1 Institute of Microbiology and Immunology, University of Belgrade Faculty of Medicine, Belgrade, Serbia; 2 HIV/AIDS Unit, Institute for Infectious and Tropical Diseases CCS, Belgrade, Serbia; 3 Virology Department, Clinical Center Serbia, Belgrade, Serbia

## 

Amino acid (aa) substitutions at position 245 of HIV-1 reverse transcriptase (RT) have been described to be associated to the presence of human leukocyte antigen (HLA)–B*5701 allele in the host, in particular in subtype B infection. Preliminary data show HLA–B*5701 prevalence of around 6.5% in Serbian population. In this study, we investigated the prevalence of RT codon 245 substitutions among HIV infected patients in Serbia. Furthermore, we analyzed the association between RT 245 aa substitutions with duration of infection, estimated by proportion of ambiguous basecalls per sequence.

The study included 184 consenting, subtype B HIV infected patients aged 18 or more. The majority of patients were newly diagnosed, 150/184, while 34/184 patients were on treatment. Pol region sequences, covering protease and minimally 250 RT codons, were obtained within routine drug resistance testing. The fraction of ambiguous nucleotides in each sequence was calculated for samples drawn from naive patients. We used ambiguity percentage of 0.47% as a cut-off value delimiting recent (less than 1 year) vs. chronic infection (longer than 1 year).

In total, predominant aa at RT codon 245 was the wild type valin (V) found in 118/184 (64.1%), hence 35.9% (66/184) contained mutation at this position (among naïve patients this percentage was 38% (57/150). The most common substitution at RT codon 245 was methionine (M) 29/184 (15.7%), followed by glutamic acid (E) 20/184 (10.9%) and others. Based on the percentage of ambiguous basecalls, a total of 55.3% of naive samples (83/150) were classified as recent infection, while among these, 57.8% (48/83) had V at position 245. A total of 45.3% (68/150) were classified as chronic infection, with the presence of V at RT codon 245 found in 69.1% (47/68). We did not find statistically significant association between polymorphisms at codon 245 and duration of infection (p=0.2080).

The frequency of RT 245 substitution found in our study exceeds the estimated prevalence HLA–B*5701 in Serbian population. This may be related to the presence of other, similar HLA alleles, limiting specificity of the correlation between HLA-B*5701 and RT codon 245 variation. Furthermore, no statistically significant difference was found in the prevalence of RT 245 substitutions between recent and chronic infection. This may suggest early fixation of an HLA induced selective imprint, during viral evolution in an infected patient.

